# Viral Infections in HSCT Recipients with Post-Transplant Lymphoproliferative Disorder: The Role of Torque Teno Virus as a Marker of Immune Functions

**DOI:** 10.3390/microorganisms13020326

**Published:** 2025-02-02

**Authors:** Martyna Pociupany, Carolina Tarabella, Robert Snoeck, Daan Dierickx, Graciela Andrei

**Affiliations:** 1 Molecular Structural and Translational Virology Research Group, Department of Microbiology, Immunology and Transplantation, Rega Institute for Medical Research, KU Leuven, 3000 Leuven, Belgium; martyna.pociupany@kuleuven.be (M.P.); robert.snoeck@kuleuven.be (R.S.); 2 Centre for Molecular and Vascular Biology, Department of Cardiovascular Sciences, KU Leuven, 3000 Leuven, Belgium; carolina.tarabella@kuleuven.be; 3 Department of Hematology, University Hospitals Leuven, 3000 Leuven, Belgium; daan.dierickx@uzleuven.be; 4 Laboratory of Experimental Hematology, Department of Oncology, KU Leuven, 3000 Leuven, Belgium

**Keywords:** post-transplant lymphoproliferative disorder, Torque Teno virus, hematopoietic stem cell transplantation, Epstein–Barr virus

## Abstract

Monitoring immune function in post-transplant patients is crucial to reduce the risk of viral infections (e.g., cytomegalovirus [CMV] or Epstein–Barr virus [EBV]), which can lead to serious complications such as post-transplant lymphoproliferative disorder (PTLD). Recently, Torque Teno virus (TTV) has attracted interest as a marker of immune function. Thus, we studied the kinetics of common post-transplant viral infections (TTV, EBV, CMV, human herpesvirus-6 [HHV-6], and adenovirus [AdV]) and their association with clinical parameters in 23 HSCT recipients who developed PTLD (PTLD-HSCT) and 25 post-HSCT patients without PTLD (Non-PTLD-HSCT) at three different timepoints: at the time of the transplant (T0), 3 months (T1), and 6 months (T2) post-HSCT. Additionally, 25 healthy donors (HD) were used as the control. EBV, CMV, HHV-6, or AdV infections were found in a few samples, while TTV was found in all of our samples. The highest TTV levels (4.61 [T0], 6.24 [T1] and 6.70 [T2] log_10_ copies/mL) were seen in PTLD-HSCT patients compared to Non-PTLD-HSCT (3.39 [T0], 4.86 [T1], and 3.75 [T2] log_10_ copies/mL) and HD (2.25 log_10_ copies/mL) at all timepoints. Higher TTV levels were also seen in patients with a destructive type of PTLD and in surviving PTLD-HSCT patients compared to deceased ones. TTV kinetics in PTLD patients post-HSCT showed that TTV levels increase with the fall in the host immunocompetence and that by monitoring TTV kinetics, the immune status of the patient can be monitored.

## 1. Introduction

The identification and management of post-transplant pathogens over recent decades have proven vital in reducing morbidity and mortality in transplant recipients [1,2]. Hematopoietic stem cell transplantation (HSCT) recipients are subjected to profound immunosuppression followed by gradual immune recovery [3,4]. The balance between maintaining high immunosuppression in the post-transplant setting to prevent graft vs. host disease (GvHD) and the emergence of post-transplant complications (relapse of the underlying condition or new primary infections) is a major hurdle for patients’ survival [3,5,6,7].

Post-transplant viral infections include those caused by herpesviruses (cytomegalovirus [CMV], herpes simplex virus [HSV], varicella–zoster virus [VZV], human herpesvirus-6 [HHV-6], Epstein–Barr virus [EBV]), or adenoviruses (AdV). These viral infections are associated with increased mortality and can lead to serious complications like EBV-driven post-transplant lymphoproliferative disorder (PTLD) [2,4,6,8,9]. This rare but life-threatening disorder, caused by heavy immunosuppressive therapy, drives the abnormal proliferation of EBV-infected B-cells [8,10,11]. Some viral co-infections, such as CMV-EBV, are considered a risk factor for developing PTLD in transplant recipients [12,13]. Thus, clinicians involved in the management of HSCT need to consider these viruses and their clinical impact.

Tools to measure immunosuppression in post-transplant patients are crucial, helping to improve patients’ prophylaxis and treatment and playing a major role in determining patients’ outcome [2,3,14]. Currently in the clinic setting, there are a few assays that can be used to measure patients’ immune system function, such as immune cell count, assessing virus-specific T-cell response, or measuring serum immunoglobulin G (IgG); however, these provide only a general estimation of patients’ immune reconstitution [14,15]. Thus, there is a fundamental need for uniform biomarkers that can be used to monitor the immunosuppression levels of patients to reduce the risk of GvHD and infections post-HSCT [5,9]. The use of standardized assays to measure patients’ immunosuppression would improve comparison between different centers [16].

Recently, Torque Teno virus (TTV) has gained interest as a possible marker of immune function [17,18]. TTV is a ubiquitous, non-enveloped single-stranded DNA virus belonging to the Anelloviridae family [3,5,6,19]. The presence of TTV is detected in around 95% of healthy individuals and in different biological samples, implying that TTV is a component of the human virome [2,5,18,20]. TTV has been linked to many various disorders; however, it still remains non-pathogenic with no specific clinical symptoms [3,7,20,21,22,23]. Moreover, TTV is not sensitive to conventional antivirals and its viral load increases when co-infection with other viruses occurs [5,7,17,18,19,20]. Since TTV is a part of the human virome tightly controlled by immune responses, its viral load would reflect the changes between high viral replication during intense immunosuppression and viral clearance during immune reconstitution [18]. With TTV representing the most abundant component of the human virome, an increase in its viral load has been observed during immunosuppression, inversely correlated with immune competence [24]. In particular, TTV was found to be inversely correlated with CD4 T-cell number and the CD4/CD8 ratio [24]. It is hypothesized that TTV replication is controlled by T cells, implying that by monitoring TTV level dynamics, the state of immunosuppression can be estimated [18,24]. Here, we employed a commercially available TTV assay (TTV R-GENE) that could be used as a standardized method for TTV quantification across multicenter clinical trials and in other investigations to enable high comparability between different study groups.

We studied the kinetics of post-transplant viral infections (TTV, EBV, CMV, HHV-6, and AdV) and their association with clinical parameters in HSCT recipients with or without PTLD at 3 different timepoints: at the time of the transplant (T0) and 3 (T1) and 6 months (T2) post-HSCT. Moreover, to our knowledge, this is the first study on TTV kinetics in PTLD patients after HSCT.

## 2. Materials and Methods

### 2.1. Study Population

For this retrospective study, 48 patients who underwent HSCT in the University Hospital of Leuven (UZ Leuven) between November 2008 and June 2018 were selected: 23 patients who developed PTLD after HSCT (PTLD-HSCT) and 25 matched patients who did not develop PTLD after HSCT (Non-PTLD-HSCT). In this retrospective study, we looked for patients post-HSCT in the 10-year timeframe who had their plasma taken at 3 different timepoints and who developed PTLD (*n* = 23), and then selected patients who, after HSCT, did not develop PTLD (*n* = 25) and also had their plasma taken at 3 different timepoints to match the number, age, and sex of patients in the PTLD group out of a bigger cohort of post-HSCT patients. Meanwhile, 25 healthy donors (HD) who did not undergo any transplant and were not diagnosed with any malignancy were also included. Demographics and hematological and transplant-related characteristics were retrieved from patients’ medical records. HD personal data were anonymous. The Ethics Committee of the UZ/KU Leuven approved the protocol before initiation of the study (S62534).

### 2.2. Collection of Human Plasma

Plasma samples were obtained from 25 HD, 23 PTLD-HSCT, and 25 Non-PTLD-HSCT patients. Plasma was obtained at three different timepoints: at the time of transplantation (T0), 3 months (T1), and 6 months (T2) after HSCT, except for HD, where plasma was obtained only once. The samples were kept at −80 °C.

### 2.3. DNA Extraction

DNA was extracted from 200 µL of plasma using the QIAamp DNA blood kit (Qiagen, Benelux BV, Venlo, the Netherlands) according to the manufacturer’s instructions.

### 2.4. Viral DNA Detection by qPCR

The quantitative determination of DNA viruses from patient DNA was performed by real-time PCR on Quant Studio7 using the R-gene kits (CMV, EBV, HHV6, TTV and Adenovirus R-Gene) (Argene, bioMérieux, Marcy-l’Étoile, Lyon, France) as recommended by the manufacturer.

### 2.5. Statistics

Calculated Cq values, melt curves, and standard curves for each target were obtained from instrument software and used for further analysis. The mean quantity of the viral load based on standard curves was log-transformed for analysis (Log copies/mL). Two-way ANOVA with the repeated measures (RM) test was used to establish significant differences in TTV levels between the 2 groups at 3 different timepoints and to establish the impact of clinical factors. One-way ANOVA with RM was used to assess the significant differences in TTV viral load and in immune cell count between the 3 timepoints in the PTLD-HSCT and Non-PTLD-HSCT groups and between the 3 groups at each timepoint. Kaplan–Meier survival curve analysis was used to create survival curves. Spearman’s correlation coefficient test was used to establish significant correlation between TTV levels and immune cell count. Significant differences in immune cell count between different timepoints were established using the Mann–Whitney test. A *p*-value of <0.05 was considered significant. Statistical analyses and data plotting were conducted using GraphPad Prism^®^ software (version 9; GraphPad software, San Diego, CA, USA).

## 3. Results

### 3.1. Participants Characteristics

A total of 23 PTLD-HSCT, 25 Non-PTLD-HSCT, and 25 HD were included in this study. PTLD diagnosis was based on biopsies, morphology examinations, and FDG-PET/CT scans. EBV association was performed histologically based on biopsy in situ hybridization of Epstein–Barr virus-encoded small RNAs (EBERs). Overall, 69.57% (16/23) of PTLD patients had positive staining for EBER, with 8.7% (2/23) of PTLD patients having a negative staining result and 5 patients (21.74%) not having a biopsy available (Table 1). When taking into account only PTLD patients with available EBER staining for their biopsies (18 patients), 88% (16/18) had a positive EBER staining and 11% (2/18) had a negative staining result.

The difference between the median age of the PTLD-HSCT group and the Non-PTLD-HSCT group was not significant (52 vs. 53 years, *p* = 0.84) (Supplementary Figure S1). There were 12 males and 11 females in the PTLD-HSCT and 16 males and 9 females in Non-PTLD-HSCT group (Supplementary Figure S1). The mean time of incidence of PTLD after HSCT was around 6–7 months; however, most patients (18/23 [78%]) were diagnosed within the first 6 months post-HSCT (Supplementary Figure S1). The most prominent underlying disease in both PTLD-HSCT and Non-PTLD-HSCT groups were myeloid malignancies (52.17% and 68%, respectively) with acute myeloid leukemia (AML) (26.09% and 48%, respectively) being the most common (Table 1). Other underlying diseases included lymphoid malignancies (21.74% in PTLD-HSCT and 20% in Non-PTLD-HSCT) and aplastic anemia or inherited disorders (Table 1). Patients and donors were checked for the presence of CMV before HSCT. The conditioning therapy was either ablative (39.13% in PTLD-HSCT and 40% in Non-PTLD-HSCT) or non-ablative (60.87% in PTLD-HSCT and 60% in Non-PTLD-HSCT). Patients’ induction therapy for the underlying disorder included chemotherapy (60.90% in PTLD-HSCT and 80% in Non-PTLD-HSCT), with the most common therapy being the use of cytarabine and anthracycline (26.10% in PTLD-HSCT and 52% in Non-PTLD-HSCT) (“3+7” approach), followed by immunosuppression (26.10%% in PTLD-HSCT and 4% in Non-PTLD-HSCT). Information about the induction treatments and conditioning methods is provided in Supplementary Table S1.

Patient demographics and clinical data including PTLD classification, viral infections found, HSCT subtype, and CMV mismatch, together with the immunosuppression strategy and the outcome of patients, are presented in Table 1.

### 3.2. Viral Infections HSCT Recipients

We first tested plasma samples for the presence of EBV, CMV, HHV-6, AdV, and TTV DNA at all 3 timepoints (Table 1). TTV DNA was detected in 100% of both PTLD-HSCT and Non-PTLD-HSCT samples at all timepoints. EBV DNA was detected in 13.04% of PTLD-HSCT samples (4 samples) in 3 patients and none in the Non-PTLD-HSCT group. CMV DNA was detected in 8.69% of PTLD-HSCT samples (2 samples) in 2 patients and in 8% of Non-PTLD-HSCT samples (3 samples) in 2 patients. HHV-6 DNA was detected in 8.70% of the PTLD-HSCT samples (3 samples) in 2 patients and in one sample (4%) from the Non-PTLD-HSCT group. Finally, AdV DNA was detected in 2 samples: in one PTLD-HSCT sample (4.35%) and one in Non-PTLD-HSCT sample (4%). The findings are described in detail in Supplementary Table S2.

### 3.3. TTV DNA Load in HSCT Recipients and Healthy Donors

**Figure 1:**
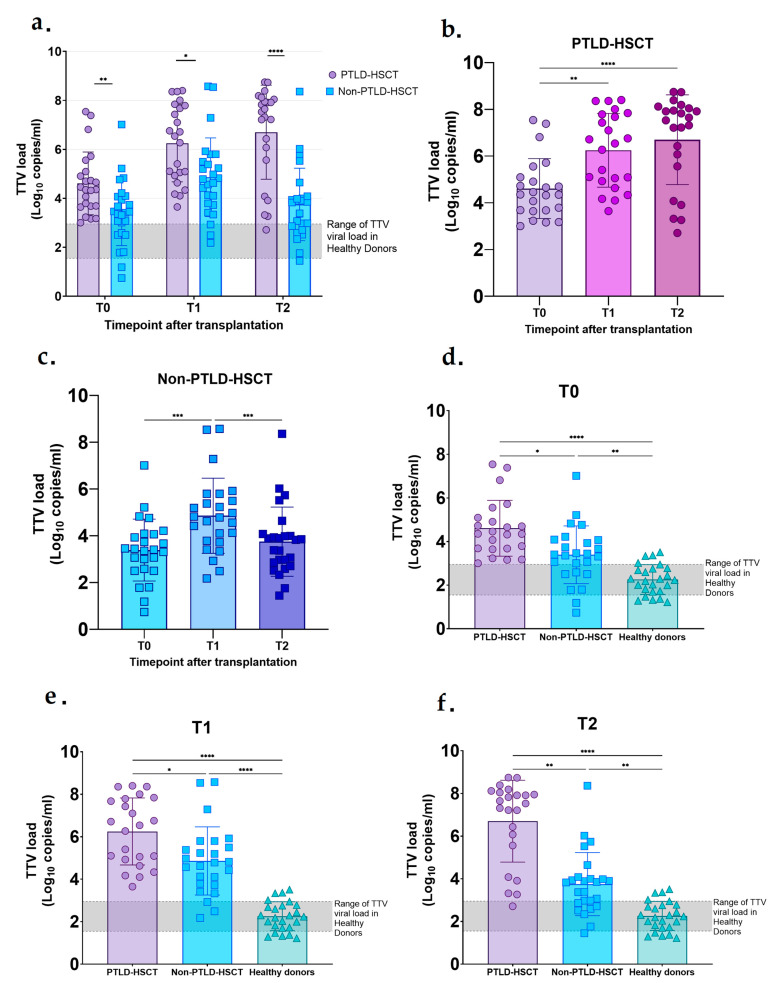
Torque Teno virus DNA load and kinetics. Viral DNA load was measured at 3 different timepoints after transplantation (T0, T1, and T2) from plasma samples of patients after HSCT who developed PTLD (purple) (PTLD-HSCT) and a control group who did not develop PTLD (blue) (Non-PTLD-HSCT), with the gray area depicting the range of TTV DNA load in healthy donors according to the literature and our data. (**a**) TTV DNA load comparison between the PTLD-HSCT and Non-PTLD-HSCT groups at different timepoints. (**b**,**c**) Kinetics of TTV DNA load throughout the timepoints (T0, T1 and T2) from plasma samples of (**b**) PTLD-HSCT and (**c**) Non-PTLD-HSCT. (**d**–**f**) TTV DNA load comparison between PTLD-HSCT, Non-PTLD-HSCT, and healthy donors (green) whose plasma sample was taken only at one timepoint, measured at (**d**) the time of transplantation (T0), (**e**) 3 months (T1), and (**f**) 6 months (T2) after transplantation. Results are presented as observed Log10 calculated based on the standard curve. Multiple groups were compared using two-way analysis of variance (ANOVA) with repeated measures (RM). One-way ANOVA with RM was used to assess differences in TTV viral load between timepoints and between groups at each timepoint. Significance was determined as a *p* value of <0.05, and the SD is shown. (*) *p* ≤ 0.05, (**) *p* < 0.01, (***) *p* < 0.001, (****) *p* < 0.0001.

Owing to the growing interest in TTV’s role as an immune function marker and the fact that we were able to detect TTV DNA in all of our samples, we decided to investigate these findings further. TTV DNA load was significantly higher in PTLD-HSCT compared to Non-PTLD-HSCT samples at all timepoints (4.61 vs. 3.39 log10 copies/mL, *p* = 0.0067 at T0; 6.25 vs. 4.86 log10 copies/mL, *p* = 0.012 at T1; 6.70 vs. 3.75 log10 copies/mL, *p* < 0.0001 at T2;) (Figure 1a). Interestingly, we could observe a steady and continuous rise in TTV levels in PTLD-HSCT samples across the timepoints (T0 vs. T1 with *p* = 0.0012; T0 vs. T2 with *p* < 0.0001 and T1 vs. T2 with *p* = ns) (Figure 1b), whereas in Non-PTLD-HSCT samples, TTV levels reached a peak at T1 and started to decrease (T0 vs. T1 with *p* = 0.0004; T0 vs. T2 with *p* = ns and T1 vs. T2 with *p* = 0.0004) (Figure 1c). We also included TTV levels detected in 25 HD, where the mean was 2.25 log copies/mL and was significantly lower compared to both PTLD-HSCT and Non-PTLD-HSCT at all timepoints (PTLD-HSCT and Non-PTLD-HSCT vs. HD with *p* < 0.0001 and *p* = 0.003 at T0, respectively; PTLD-HSCT and Non-PTLD-HSCT vs. HD with both *p* < 0.0001 at T1; PTLD-HSCT and Non-PTLD-HSCT vs. HD with *p* < 0.0001 and *p* = 0.002 at T2, respectively;) (Figure 1d,e).

### 3.4. Correlation Between TTV DNA Load and Immune Cells

**Figure 2:**
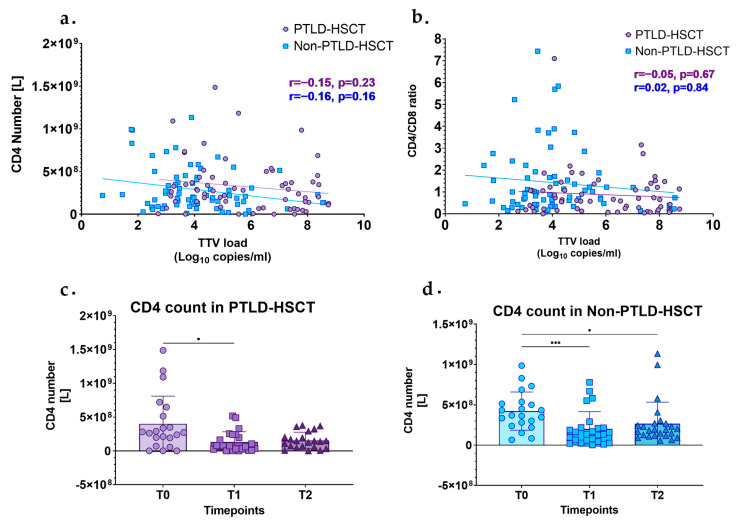
Correlation between Torque Teno virus DNA load and immune cell count. (**a**) TTV DNA load correlation with CD4 count in the PTLD-HSCT (purple) and Non-PTLD-HSCT 8 (blue) group at three timepoints. (**b**) Correlation between CD4/CD8 ratio and TTV levels in the PTLD-HSCT (purple) and Non-PTLD-HSCT (blue) groups at the three timepoints. (**c**,**d**) Comparison between CD4 counts in the (**c**) PTLD-HSCT and (**d**) Non-PTLD-HSCT groups across the timepoints. The Spearman correlation coefficient (r) test was used for correlations. The Mann–Whitney test was used to establish differences in cell count between timepoints. Significance was determined as a *p* value of <0.05. (*) *p* ≤ 0.05, (***) *p* < 0.001.

We also correlated TTV levels with immune cell count and with the CD4/CD8 ratio (Figure 2a,b). We found no correlation between CD4 T-cell count or CD8 T-cell count and TTV DNA load at any timepoint (Supplementary Figure S2). We observed an association of decreased CD4 T-cell count and lower CD4/CD8 ratio with higher TTV DNA load; however, this correlartion was not significant (Figure 2a,b). TTV levels increase with a decrease in CD4 T-cell count, as indicated by the significant decline in CD4 number from T0 to T1 in the PTLD-HSCT group (T0 = 4 × 10^8^ vs. T1 = 1.32 × 10^8^, *p* = 0.016). We also observed a significant decrease in CD4 T-cell count in the Non-PTLD-HSCT group from T0 to T1 (T0 = 4.2 × 10^8^ vs. T1 = 2 × 10^8^, *p* = 0.0004) and a slight increase from T1 to T2 following the changes in TTV levels (i.e., an increase from T0 to T1 and a decrease from T1 to T2) (Figure 2c,d). The correlations of CD4 and CD8 T-cell counts and TTV DNA load at each timepoint and the difference in immune cell count between the PTLD-HSCT and Non-PTLD-HSCT groups across the timepoints are presented in Supplementary Figures S2 and S3.

### 3.5. Correlation Between TTV DNA Load and Transplant Characteristics

**Figure 3:**
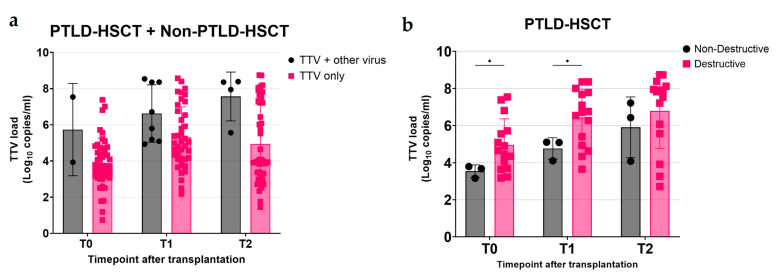
TTV DNA load in HSCT recipients categorized by (post-)transplant viral infection/reactivation and PTLD classification. (**a**) Comparisons between TTV DNA load from 2, 8, and 4 PTLD-HSCT and Non-PTLD-HSCT patients combined with viral co-infection (black) and 46, 40, or 44 without viral opportunistic infection/reactivation (pink) at T0, T1, and T2, respectively, are presented. (**b**) TTV DNA load from 3 PTLD patients with non-destructive PTLD (black) and 15 with destructive PTLD (pink) at T0, T1, and T2, respectively. Comparison between groups was performed using two-way analysis of variance (ANOVA). Significance was determined as a *p* value of <0.05 and SD is shown. (*) *p* ≤ 0.05.

Firstly, we investigated the impact of viral infections on TTV DNA load, since other studies reported higher TTV levels with co-infection with other viruses [17]. We combined the PTLD-HSCT and Non-PTLD-HSCT groups to see the impact of viral co-infections on TTV DNA load in HSCT recipients overall. The mean of TTV levels with viral opportunistic infections at all timepoints was not significantly different (also due to the small number of co-infections detected), but higher than that without viral co-infections (5.73 vs. 3.89 log10 copies/mL, *p* = 0.86 at T0; 6.62 vs. 5.30 log10 copies/mL, *p* = 0.16 at T1; 7.56 vs. 4.94 log10 copies/mL, *p* = 0.059 at T2) (Figure 3a).

Next, we wanted to assess the impact of clinical factors (age, gender, hematopoietic progenitor cell source, underlying disorder, PTLD classification, CMV status, and ablative conditioning) on TTV levels. We found no statistically relevant correlations between clinical factors (age, gender, hematopoietic progenitor cell source, underlying disorder, CMV status, or ablative conditioning) and TTV levels in PTLD-HSCT or Non-PTLD-HSCT (Supplementary Figures S4–S8).

We observed a correlation between TTV levels and the PTLD type. Patients with a destructive type of PTLD had a significantly higher mean of TTV DNA load at T0 and T1 when compared to patients with non-destructive PTLD (4.95 vs. 3.54 log10 copies/mL, *p* = 0.01 at T0; 6.43 vs. 4.76 log10 copies/mL, *p* = 0.03 at T1) (Figure 3b). The difference in TTV DNA load at T2 was not significant (6.79 vs. 5.91 log10 copies/mL, *p* = 0.84 at T2); however, TTV levels in patients with destructive PTLD were higher compared to patients with non-destructive PTLD.

### 3.6. Outcome in PTLD-HSCT and CONTR-HSCT

Lastly, we investigated the impact of TTV DNA load on the outcome of post-HSCT patients according to the patient state at last follow-up date. We categorized patients as alive (A) or deceased (D) at last follow-up date.

**Figure 4:**
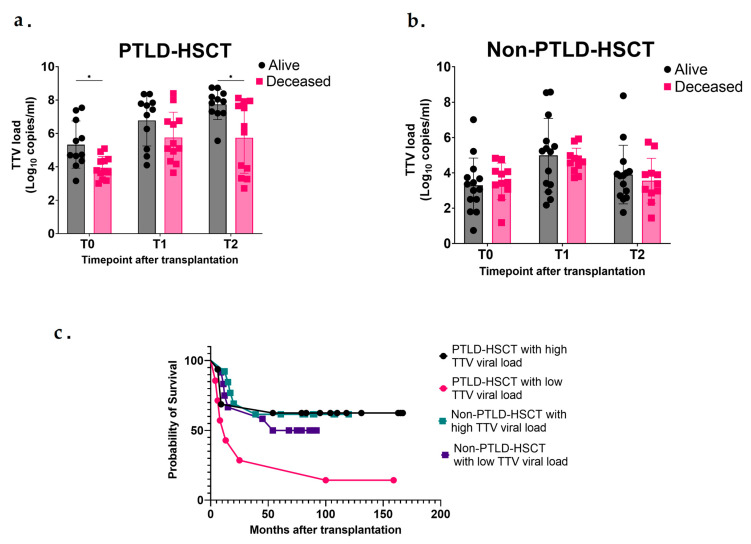
Impact of TTV DNA load in HSCT recipients on patient outcome. TTV DNA loads from (**a**) 11 alive PTLD-HSCT patients (black) and 12 deceased (pink) PTLD-HSCT patients and (**b**) 14 alive Non-PTLD-HSCT patients (black) and 11 deceased (pink) Non-PTLD-HSCT patients are presented. (**c**) Kaplan–Meier survival plot of HSCT recipients with TTV DNA load stratified by high or low load. Kaplan–Meier curve displaying estimated probability of survival of HSCT recipients (PTLD-HSCT and Non-PTLD-HSCT). Comparison between groups was performed using two-way analysis of variance (ANOVA) with repeated measures. Significance was determined as a *p* value of <0.05, and the SD is shown. (*) *p* ≤ 0.05.

In the PTLD-HSCT group, higher levels of TTV were seen in alive patients at all timepoints compared to deceased patients and the difference was statistically significant at T0 and T2 (5.33 vs. 3.94 log10 copies/mL, *p* = 0.03 at T0; 6.78 vs. 5.75 log10 copies/mL, *p* = 0.32 at T1; 7.74 vs. 5.74 log10 copies/mL, *p* = 0.02 at T2) (Figure 4a). Interestingly, when assessing TTV levels’ impact on patient outcome in the Non-PTLD-HSCT group, we could not see any significant difference between alive and deceased patients, where the TTV DNA load was similar at all timepoints (3.29 vs. 3.52 log10 copies/mL, *p* = 0.96 at T0; 4.99 vs. 4.68 log10 copies/mL, *p* = 0.94 at T1; 3.90 vs. 3.55 log10 copies/mL, *p* = 0.91 at T2) (Figure 4b).

Additionally, we produced Kaplan–Meier curves which were stratified by TTV levels (high or low TTV DNA load based on the mean of TTV levels) in PTLD-HSCT and in Non-PTLD-HSCT (Figure 4c). Higher TTV DNA load was associated with better survival in the PTLD-HSCT group (*p* = 0.002), whereas in the Non-PTLD-HSCT group, there was no difference in the survival of patients with a high or low level of TTV.

In conclusion, we investigated the human virome in post-HSCT recipients who either developed or did not develop PTLD. We found only a few EBV, CMV, HHV-6, and AdV infections. A TTV DNA load was detected in all samples and was significantly higher in PTLD-HSCT compared to Non-PTLD-HSCT and HD. TTV levels in PTLD-HSCT kept rising from T0 to T2, whereas in Non-PTLD-HSCT, the DNA load peaked at T1. We found no correlation between TTV levels and age, gender, HPC source, underlying disorder, or CMV status (Tables S3–S5). Interestingly, TTV levels were significantly higher in PTLD patients with a destructive PTLD type and surviving patients with PTLD compared to deceased patients with PTLD at the last follow up date.

## 4. Discussion

Viruses belonging to the Herpesviridae family and their primary infections or reactivations have been associated with severe complications among HSCT recipients [1,25]. Zanella et al. investigated the presence of DNA and RNA viruses in HSCT recipients, where they found prevalent TTV infections in 97% of patients, CMV in up to 27% of patients, often co-detected with HHV-6, and EBV’s presence in below 5% of patients [25]. Defining the landscape of viral infections or reactivations after transplantation is crucial, since they can cause serious complications like PTLD in post-transplant patients. Therefore, we investigated common post-transplant infections in HSCT recipients with and without PTLD. We found few EBV, CMV, HHV-6, and AdV infections in plasma samples. Plasma is a cell-free specimen, and thus only genetic material from viruses in the lytic stage (during active infection/reactivation) can be found. Compared to plasma, whole-blood samples have a higher DNA load and the viral DNA can be detected in a larger number of patients [26,27].

In our study, in line with previous reports, TTV levels were significantly higher in HSCT recipients compared to HD [3,4,9,18,28,29]. Focosi et al. examined TTV’s presence in 1017 plasma samples of healthy volunteers and concluded that the mean TTV load was 2.3 ± 0.7 Log copies/mL and remained stable for 2 years, which is in line with our healthy donor TTV DNA load mean, reaching 2.25 ± 0.68 Log copies/mL [29].

The peak of TTV levels is usually seen at 80–100 days post-HSCT, followed by a plateau [3,18,30,31]. Wohlfarth et al. screened plasma samples for TTV every month up to a year post-transplantation, with the peak of TTV levels seen at day 79 and TTV viremia being sustained through the first year post-HSCT [20]. Similarly, other studies report the peak of TTV levels at around the 100th day post-HSCT [7]. In our investigation, TTV levels kept rising from T0 to T2 (160–180 days post-HSCT) in PTLD-HSCT group, and in the Non-PTLD-HSCT group, the DNA load peaked at T1 (80–100 days post-HSCT) and then decreased. The most efficient timepoint for TTV levels to be used as a potential biomarker seems to be around day 100 (T1), when TTV DNA load reaches its peak and starts to be affected by immune reconstitution [4,7,9].

The relationship between TTV and other viruses remains controversial [32]. It was suggested that TTV chronic infection promotes neoplastic development in association with EBV [33]. Borkosky et al. proposed that EBV acts as a helper virus and stimulates TTV replication [34]. However, the association between EBV and TTV is still not proven [35]. A link between TTV and CMV levels in HSCT recipients was also reported [9,30]. However, other reports saw no correlations between TTV and CMV levels but proposed the possibility of predicting patients at risk of developing high CMV DNAemia by analyzing TTV kinetics shortly after HSCT [36,37]. Additionally, increased TTV levels in HSCT recipients were associated with CMV and EBV reactivation, but due to various cofounding factors, TTV load is not predictive of clinical complications [3,7,20].

Lower TTV levels reflect higher functions of the immune system based on lower incidence of CMV and EBV infections, while higher TTV levels are seen in HSCT recipients with viral co-infections [4]. In our study, due to the small number of co-infections found, we did not see any significant results; however, we observed a trend where higher TTV levels were seen when a patient was co-infected with another virus.

The current state of knowledge suggests that TTV is a component of the human virome since TTV DNA can be detected in the blood, plasma, cord blood, or PBMCs [1,2]. Owing to its distinctive characteristics, TTV can serve as a unique tool to measure immune function [2]. Few studies have investigated the role of TTV as a potential marker of immune function in HSCT, and these found that TTV levels could serve as a marker of immune reconstitution after transplant [30,31,36]. Our study provided TTV dynamics after HSCT at different timepoints and showed that TTV could be used as a marker for immunosuppression and immune reconstitution.

Previously, it was shown that immunosuppressive therapy post-transplantation or CD4 T-cell depletion due to human immunodeficiency virus (HIV) infection leads to an increase in TTV levels, which also correlates with an immunosuppressive state [38]. Lower CD4 T-cell count was associated with higher TTV levels in previous reports as well as in our analysis [24,38,39]. An inverse correlation between TTV DNA load, CD4 T-cells, and CD4/CD8 ratio in immunosuppressed patients was also described previously [38,39]. We did not observe any significant correlation between the immune cell count or CD4/CD8 ratio and TTV levels; however, we did see an association between lower CD4 T-cell count and lower CD4/CD8 ratio and higher TTV levels, suggesting that TTV might be used as a marker of immune function in post-HSCT patients [3,24,38].

When comparing clinical factors to TTV levels, we found an association between TTV DNA load and PTLD classification and patient outcome. Higher TTV DNA load was observed in alive PTLD patients at the last follow-up date when compared to deceased patients at all timepoints, which is not reflected by our results in the Non-PTLD-HSCT group. Since PTLD patients need further immunosuppressive treatment, we hypothesized that the higher TTV levels in alive patients could be explained by an effective reaction to this treatment. However, more extensive research needs to be conducted to fully explain the reasons behind this correlation. Additionally, due to the potential impact of cofounding variables, the correlation between TTV DNA load in PTLD patients and their outcome needs to be addressed in future prospective studies.

Our work represents the first investigation of TTV level kinetics in PTLD patients post-HSCT. Currently, there is no widely standardized TTV diagnostic method; however, in our study, we employed a commercially available TTV assay (TTV R-GENE) that could be used to standardize methods of TTV quantification across multi-center clinical trails and other investigations. Our study, however, has also few limitations, like the size of our cohort and the retrospective nature of the study, meaning that it depends on reviews available clinical information (not initially designed to collect data for research). Nonetheless, we believe that our investigation can warrant future multi-center investigations to evaluate the role of TTV as a marker of immunocompetence in HSCT recipients and the role of TTV levels as a predictive factor for developing PTLD post-HSCT.

Our novel study of TTV kinetics post-HSCT demonstrated the highest levels of TTV in immunocompromised PTLD patients and the lowest in healthy donors, suggesting that TTV levels increase with a reduction in the host’s immunocompetence. Moreover, the monitoring of TTV kinetics after transplantation could potentially be used to tailor immune suppression.

## Figures and Tables

**Table 1:** Characteristics of hematopoietic stem cell transplant recipients.

	PTLD-HSCT n = 23	Non-PTLD-HSCT n = 25
Demographics		
Age, median (range)	52 (16–67)	53 (0–68)
Sex (n)	M (12); F (11)	M (16); F (9)
Average time from HSCT to PTLD diagnosis		
Average time to PTLD diagnosis in months (range)	6.7 (1–54)	
CMV status (n) %		
CMV mismatch	6 (26.10%)	8 (32.00%)
Donor-positive/Recipient-negative [CMV D+/R−]	4 (17.40%)	2 (8.00%)
Donor-negative/Recipient-positive [CMV D−/R+]	2 (8.70%)	6 (24.00%)
CMV match	4 (17.40%)	15 (60.00%)
Donor-negative/Recipient-negative [CMV D−/R−]	3 (13.05%)	7 (28.00%)
Donor-positive/Recipient-positive [CMV D+/R+]	1 (4.35%)	8 (32.00%)
Not available	13 (56.50%)	2 (8.00%)
Viral infections		
Torque Teno virus (TTV) infection	23 (100%)	25 (100%)
Epstein–Barr virus (EBV) infection	3 (13.05%)	
Cytomegalovirus (CMV) infection	2 (8.70%)	2 (8.00%)
Human herpesvirus-6 (HHV-6) infection	2 (8.70%)	1 (4.00%)
Adenovirus (AdV) infection	1 (4.35%)	1 (4.00%)
Hematopoietic progenitor cell source (HPC) (n) %		
HPC from apheresis (HPC-A)	18 (78.26%)	22 (88.00%)
HPC from bone marrow (HPC-M)	5 (21.74%)	3 (12.00%)
HSCT subtype (n) %		
Matched unrelated donor (MUD)	16 (69.57%)	11 (44.00%)
Matched related donor (MRD)	4 (17.39%)	11 (44.00%)
Haploidentical (Haplo-id)	3 (13.05%)	3 (12.00%)
Umbilical cord blood (UCB)	1 (4.35%)	
Underlying disorder (n) %		
Lymphoid malignancies (L)	5 (21.74%)	5 (20.00%)
Hodgkin lymphoma (HL)	1 (4.35%)	2 (8.00%)
T-cell/NK-cell lymphoma	2 (8.70%)	1 (4.00%)
Mantle cell lymphoma (MCL)	1 (4.35%)	
Acute lymphoblastic leukemia (ALL)	1 (4.35%)	2 (8.00%)
Myeloid malignancies (M)	12 (52.14%)	17 (68.00%)
Acute myeloid leukemia (AML)	6 (26.09%)	12 (48.00%)
Myelodysplastic syndrome (MDS)	2 (8.70%)	3 (12.00%)
Myelofibrosis	1 (4.35%)	
Chronic myelogenous leukemia (CML)	1 (4.35%)	
Multiple Myeloma (MM)		1 (4.00%)
Others	2 (8.70%)	3 (12.00%)
Aplastic anemia (AA)	2 (8.70%)	1 (4.00%)
Inherited disorders		2 (8.00%)
Not available	6 (26.09%)	
GvHD prophylaxis (n) %		
ATG^1^, CsA^2^ and MTX^3^	5 (21.74%)	2 (8.00%)
ATG, CsA and MMF^4^	1 (4.35%)	
CsA and MMF	2 (8.70%)	2 (8.00%)
MMF	1 (4.35%)	
CsA		2 (8.00%)
CsA and CP^5^		3 (12.00%)
MMF and Prograft		1 (4.00%)
CsA, MMF and Prograft		1 (4.00%)
ATG and MTX		1 (4.00%)
Not available	14 (60.86%)	3 (12.00%)
Ablative conditioning (n) %		
Yes	9 (39.13%)	10 (40.00%)
No	14 (60.87%)	15 (60.00%)
Induction treatment (n) %		
Chemotherapy	14 (60.87%)	20 (80.00%)
Immunosuppression	6 (26.09%)	1 (4.00%)
Other	2 (8.70%)	
No previous therapy	1 (4.35%)	4 (16.00%)
PTLD classification (n) %		
Non-destructive	3 (13.04%)	
Plasmatic hyperplasia	3 (13.04%)	
Destructive	15 (65.22%)	
Monomorphic	11 (47.83%)	
Diffuse Large B-cell Lymphoma (DLBCL)	10 (43.48%)	
High-Grade B-Cell Lymphoma (HGBCL)	1 (4.35%)	
Polymorphic	4 (17.39%)	
Plasmablastic lymphoma (PBL)-PTLD	4 (17.39%)	
No biopsy	5 (21.74%)	
In situ hybridization of EBER from biopsies of PTLD patients		
Positive	16 (69.57%)	
Negative	2 (8.70%)	
Not available	5 (21.74%)	
Outcome at last follow up date (n) %		
Alive	11 (47.83%)	14 (56.00%)
Dead	12 (52.17%)	11 (44.00%)

ATG^1^—anti-thymocyte globulin; CsA^2^—cyclosporine A; MTX^3^—methotrexate; MMF^4^—mycophenolate mofetil; CP^5^—cyclophosphamide.

## Data Availability

The original contributions presented in the study are included in the article/Supplementary Materials; further inquiries can be directed to the corresponding author.

## Supplementary Materials

The following supporting information can be downloaded at: https://www.mdpi.com/article/10.3390/microorganisms13020326/s1, Figure S1: Comparison of patient characteristics between PTLD-HSCT and Non-PTLD-HSCT; Figure S2: Correlations of immune cell count and TTV DNA load at different timepoints; Figure S3: Comparison of immune cell count between the PTLD-HSCT and Non-PTLD-HSCT groups; Figure S4: Impact of age and gender on TTV DNA load in HSCT recipients; Figure S5: Impact of CMV status on TTV DNA load in HSCT recipients; Figure S6: Impact of HSCT characteristics on TTV DNA load in HSCT recipients; Figure S7: Impact of underlying disease type on TTV DNA load in HSCT recipients; Figure S8: Impact of ablative conditioning on TTV DNA load in HSCT recipients; Table S1: Conditioning and induction treatment in post-transplant patients; Table S2: Viral DNA found in plasma samples of PTLD-HSCT and Non-PTLD-HSCT; Table S3: Multiple factors influencing TTV DNA load in PTLD patients after HSCT; Table S4: Multiple factors influencing TTV DNA load in Non-PTLD patients after HSCT; Table S5: Multiple factors influencing TTV DNA load in HSCT recipients.
