# Supplementary material for: Viral Infections in HSCT Recipients with Post-Transplant Lymphoproliferative Disorder: The Role of Torque Teno Virus as a Marker of Immune Functions
Source: Microorganisms. 2025 Feb 2;13(2):326. doi: 10.3390/microorganisms13020326 (PMC11857982; doi:10.3390/microorganisms13020326)
Supplement: Supplementary file 1 [file microorganisms-13-00326-s001.zip › Supplementary Figures.pdf]

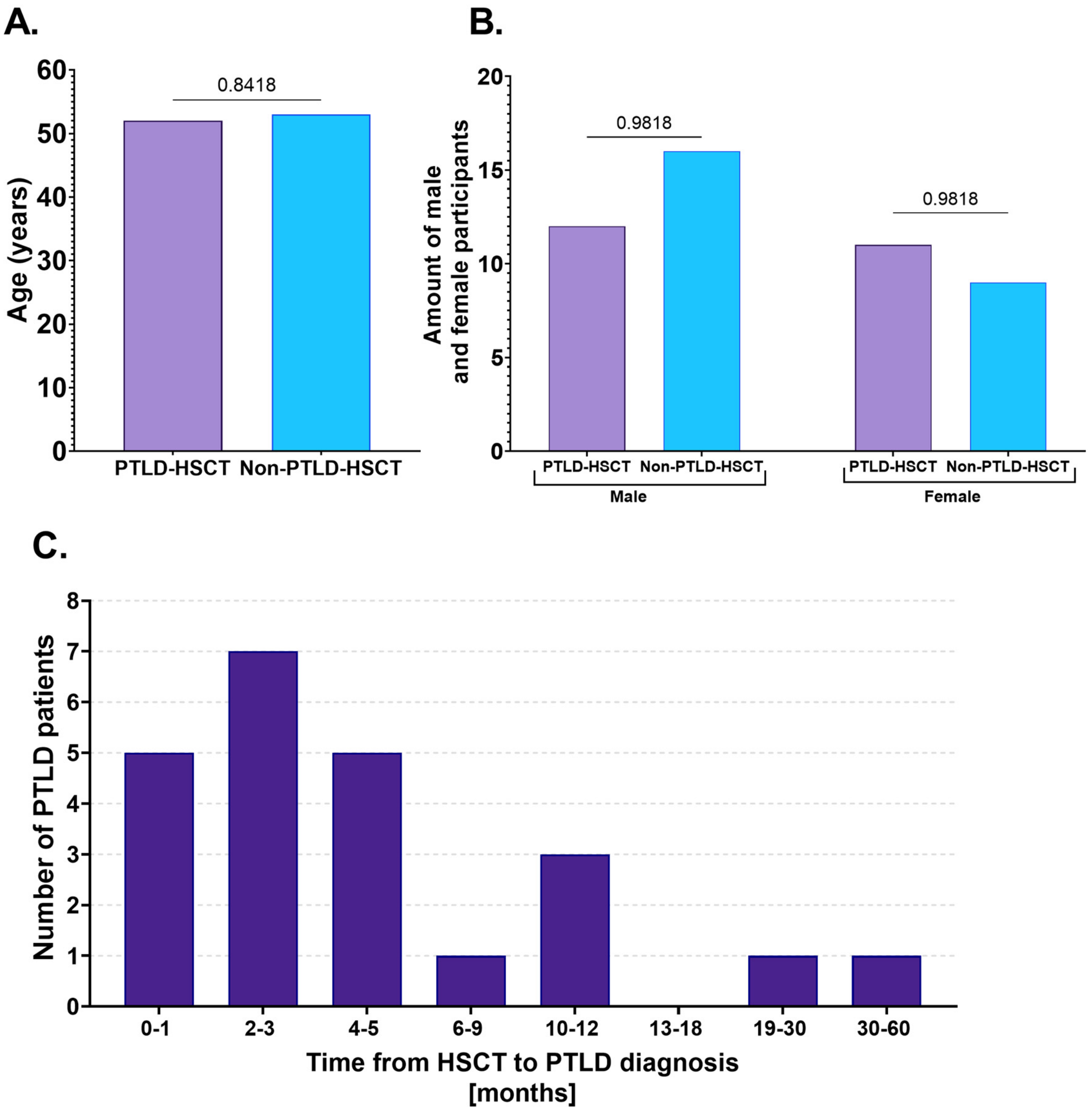

Supplementary Figure S1. Comparison of patients characteristics between PTLD-HSCT and Non-PTLD-HSCT. The difference in age **(A)** between median of PTLD-HSCT (52) and Non-PTLD-HSCT (53) is not significant (p=0.84). **(B)** The PTLD-HSCT included 12 males and 11 females whereas the Non-PTLD-HSCT cohort included 16 males and 9 females. **(C)** Graph bar showing the time and number of PTLD-HSCT patients diagnosed with PTLD post-HSCT presented in months.

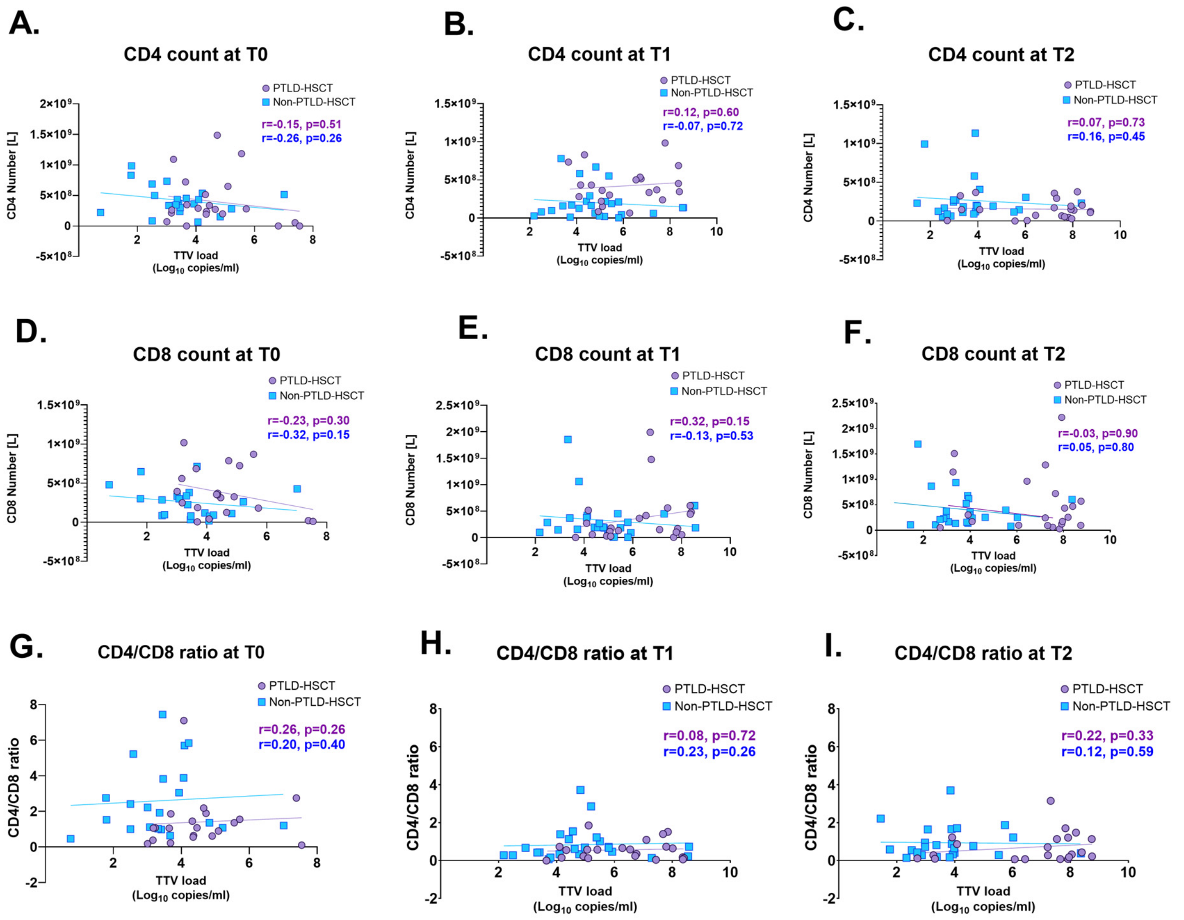

Supplementary Figure S2. Correlations of immune cells count and TTV DNA load at different timepoints. TTV DNA load correlation with **(A-C)** CD4, **(D-F)** CD8 count and **(G-I)** CD4/CD8 ratio in PTLD-HSCT and Non-PTLD-HSCT group at **(A,D,G)** T0, **(B,E,H)** T1 and **(C,F,I)** T2.The Spearman correlation coefficient (r) test was used for correlations. Significance was determined as a P value of <0.05

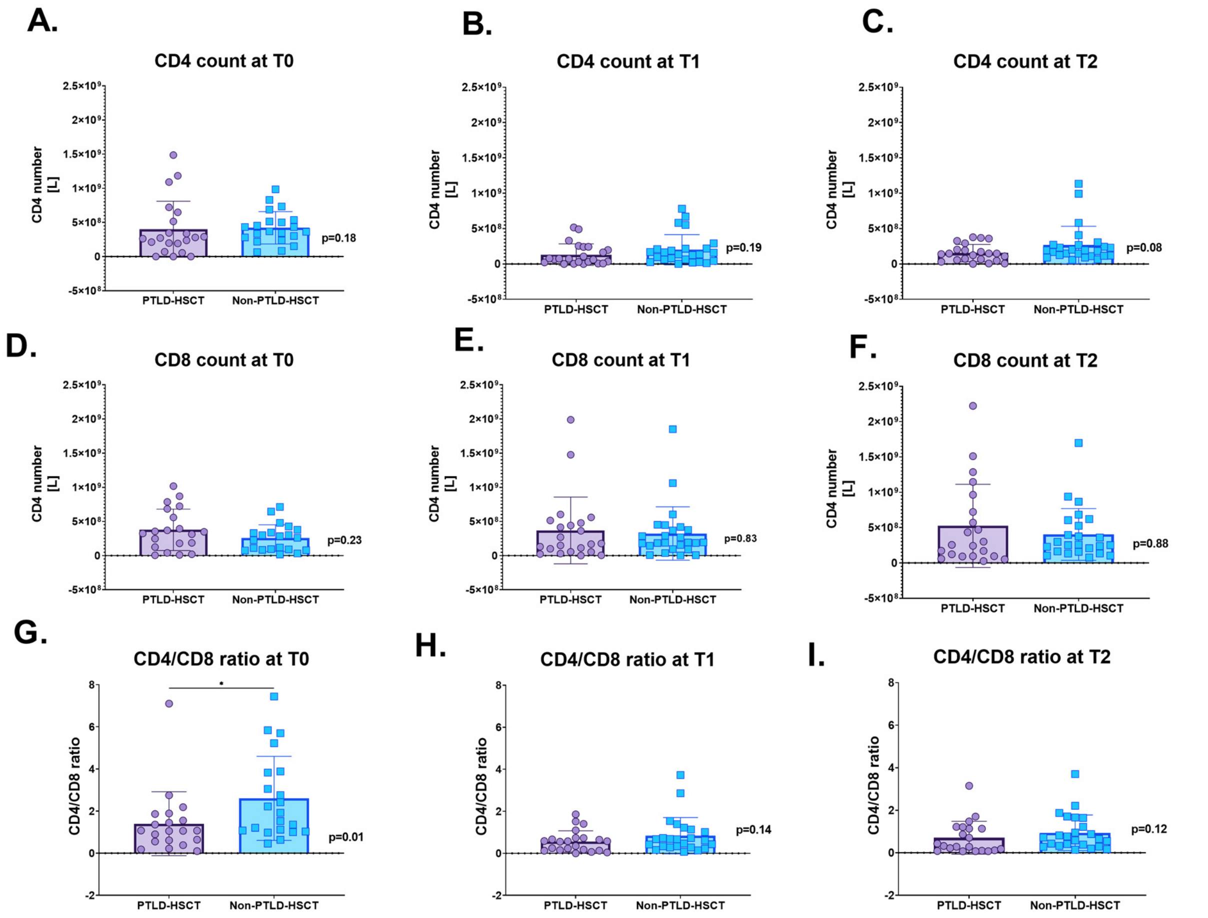

Supplementary Figure S3. Comparison of immune cell count between PTLD-HSCT and Non-PTLD-HSCT groups. Comparison of **(A-C)** CD4, **(D-F)** CD8 count and **(G-I)** CD4/CD8 ratio between PTLD-HSCT and Non-PTLD-HSCT group at **(A,D,G)** T0, **(B,E,H)** T1 and **(C,F,I)** T2. Mann-Whitney test was used to establish differences in cell count between timepoints. Significance was determined as a P value of <0.05

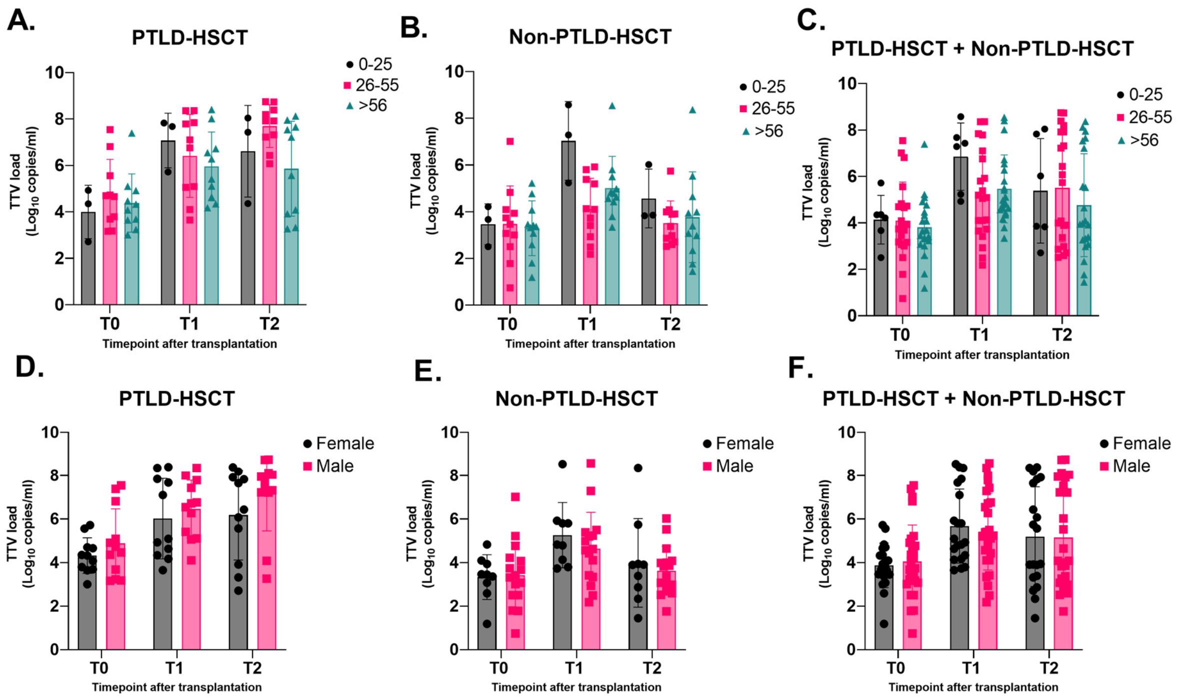

Supplementary Figure S4. Impact of age and gender on TTV DNA load in HSCT recipients. TTV DNA load depending on **(A-C)** age in **(A)** PTLD-HSCT, **(B)** Non-PTLD-HSCT, **(C)** in both PTLD-HSCT and Non-PTLD-HSCT and based on **(D-F)** gender in **(D)** PTLD-HSCT, **(E)** Non-PTLD-HSCT and **(F)** in both PTLD-HSCT and Non-PTLD-HSCT. Comparison between groups was done using two-way analysis of variance (ANOVA) with repeated measures. Significance was determined as a P value of <0.05.

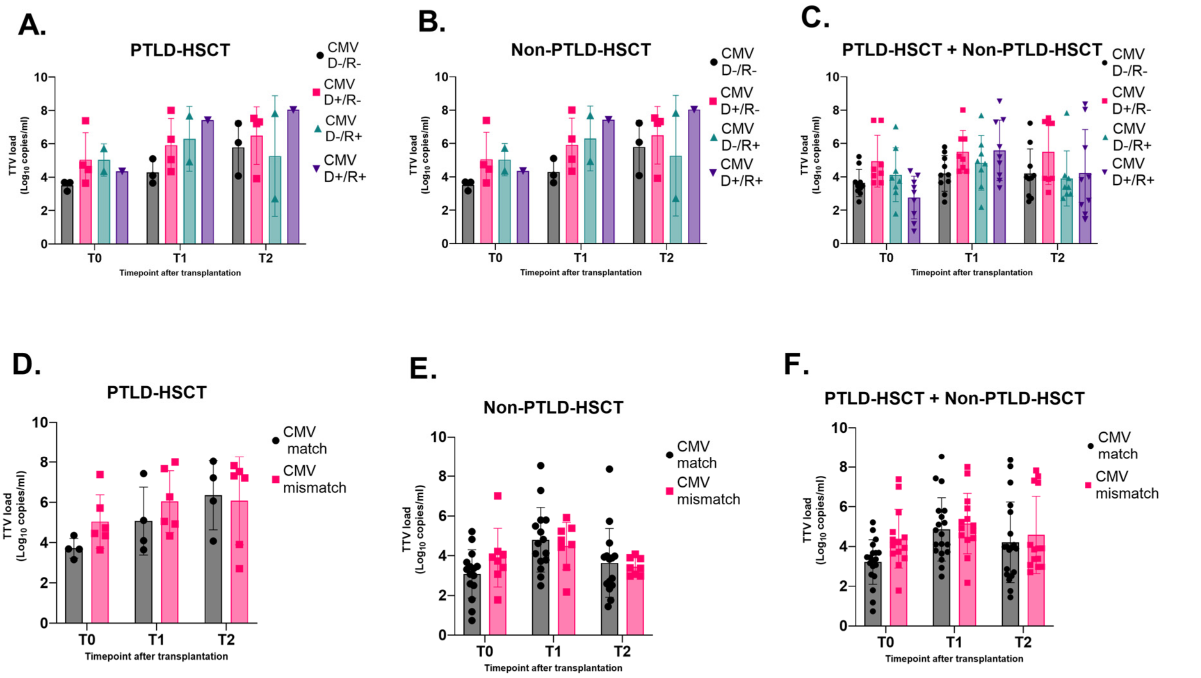

Supplementary Figure S5. Impact of CMV status on TTV DNA load in HSCT recipients. TTV DNA load depending on **(A-C)** CMV status of donor (D) and recipient (R) in **(A)** PTLD-HSCT, **(B)** Non-PTLD-HSCT, **(C)** in both PTLD-HSCT and Non-PTLD-HSCT and based on **(D-F)** CMV mismatch in **(D)** PTLD-HSCT, **(E)** Non-PTLD-HSCT and **(F)** in both PTLD-HSCT and Non-PTLD-HSCT. Comparison between groups was done using two-way analysis of variance (ANOVA) with repeated measures. Significance was determined as a P value of <0.05.

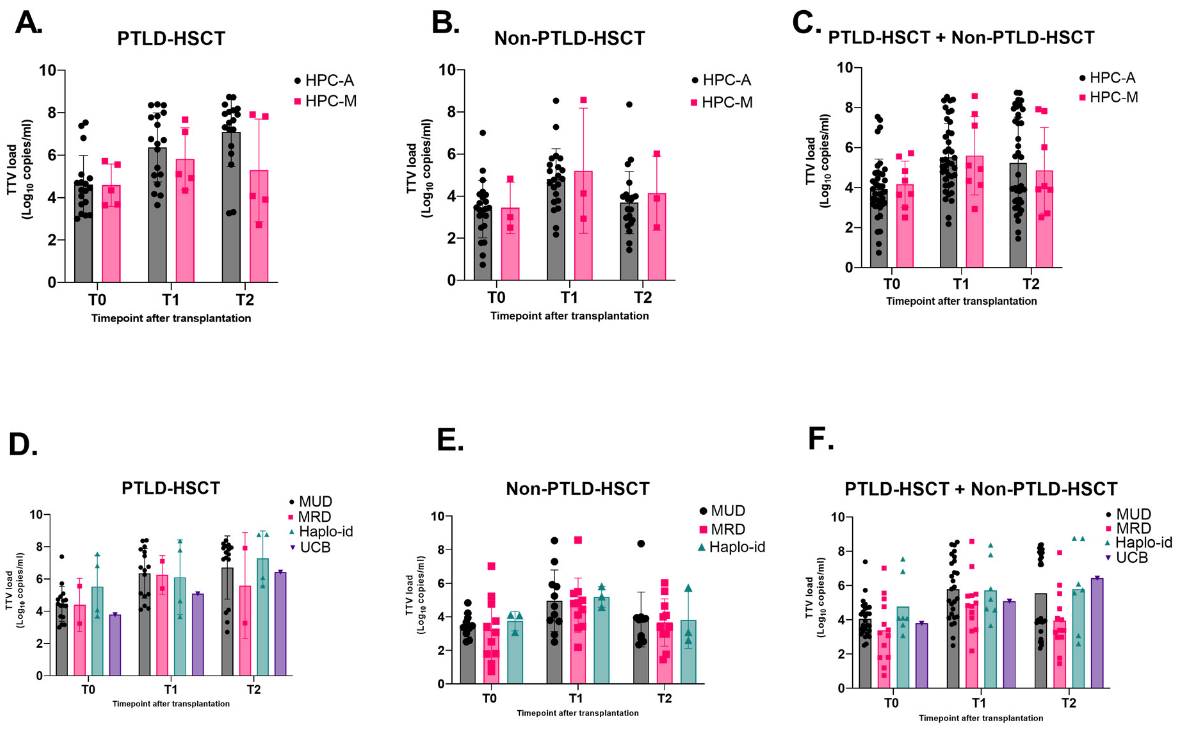

Supplementary Figure S6. Impact of HSCT characteristics on TTV DNA load in HSCT recipients. TTV DNA load depending on **(A-C)** the source of hematopoietic stem cells (Hematopoietic progenitor cells [HPC] from apheresis [HPC-A]) or from bone marrow [HPC-M]) in **(A)** PTLD-HSCT, **(B)** Non-PTLD-HSCT, **(C)** in both PTLD-HSCT and Non-PTLD-HSCT and based on **(D-F)** HSCT subtype (matched-unrelated-donor [MUD], matched-related-donor [MRD], Haploidentical hematopoietic stem cell transplantation [haplo-id] or Umbilical cord blood [UCB]) in **(D)** PTLD-HSCT, **(E)** Non-PTLD-HSCT and **(F)** in both PTLD-HSCT and Non-PTLD-HSCT. Comparison between groups was done using two-way analysis of variance (ANOVA) with repeated measures. Significance was determined as a P value of <0.05.

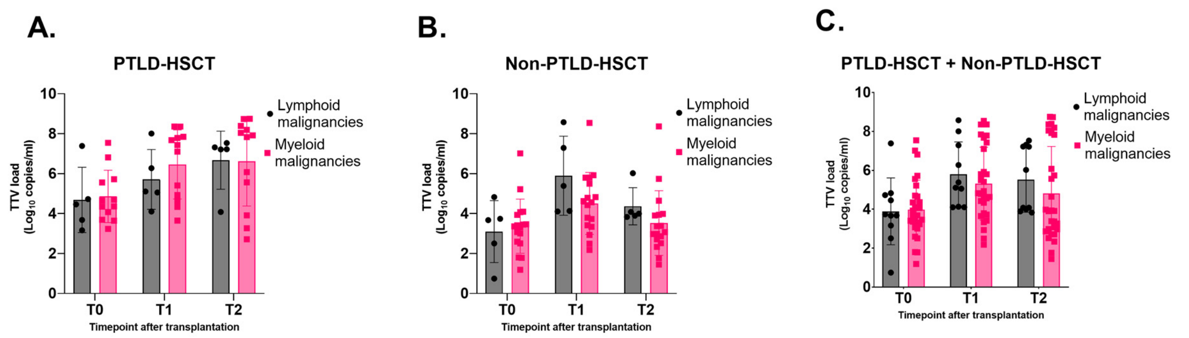

Supplementary Figure S7. Impact of underlying disease type on TTV DNA load in HSCT recipients. TTV DNA load depending on **(A-C)** the type of underlying disease in **(A)** PTLD-HSCT, **(B)** Non-PTLD-HSCT, **(C)** in both PTLD-HSCT and Non-PTLD-HSCT. Comparison between groups was done using two-way analysis of variance (ANOVA) with repeated measures. Significance was determined as a P value of <0.05.

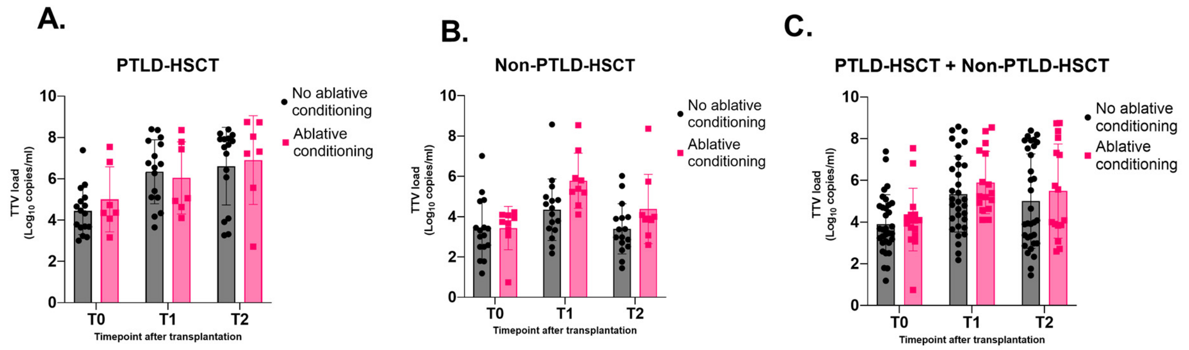

Supplementary Figure S8. Impact of ablative conditioning on TTV DNA load in HSCT recipients. TTV DNA load depending on **(A-C)** if ablative conditioning was introduced in **(A)** PTLD-HSCT, **(B)** Non-PTLD-HSCT, **(C)** in both PTLD-HSCT and Non-PTLD-HSCT. Comparison between groups was done using two-way analysis of variance (ANOVA) with repeated measures. Significance was determined as a P value of <0.05.
