# Supplementary material for: Viral Infections in HSCT Recipients with Post-Transplant Lymphoproliferative Disorder: The Role of Torque Teno Virus as a Marker of Immune Functions
Source: Microorganisms. 2025 Feb 2;13(2):326. doi: 10.3390/microorganisms13020326 (PMC11857982; doi:10.3390/microorganisms13020326)
Supplement: Supplementary file 1 [file microorganisms-13-00326-s001.zip › Table S1.pdf]

Supplementary Table S1. Conditioning and induction treatment in post-transplant patients

	PTLD-HSCT n=23	Non-PTLD-HSCT n=25
**Ablative conditioning (n) %**		
**Yes**	**9 (39.13%)**	**10 (40.00%)**
Total Body Irradiation (TBI) and cyclophosphamide	3 (13.04%)	3 (12.00%)
TBI, fludarabine and thiotepa	1 (4.35%)	
TBI, fludarabine and cyclophosphamide	1 (4.35%)	1 (4.00%)
Fludarabine and thiotepa	1 (4.35%)	
Fludarabine, melphalan and thiotepa	3 (13.04%)	1 (4.00%)
Fludarabine and treosulfan		1 (4.00%)
TBI, melphalan and thiotepa		
Fludarabine, thiotepa and treosulfan		1 (4.00%)
Fludarabine, cyclophosphamide and busulfan		2 (8.00%)
Fludarabine and busulfan		1 (4.00%)
**No**	**14 (60.87%)**	**15 (60.00%)**
Fludarabine and busulfan	11 (47.83)	9 (36.00%)
Fludarabine and cyclophosphamide	3 (13.04%)	1 (4.00%)
Fludarabine		1 (4.00%)
Fludarabine, cyclophosphamide and TBI single fraction		3 (12.00%)
Fludarabine, cyclophosphamide and busulfan		1 (4.00%)
**Consolidation method (n) %**		
**Chemotherapy**	**14 (60.90%)**	**20 (80.00%)**
Cytarabine and anthracycline	6 (26.10%)	13 (52.00%)
Cytarabine, anthracycline and ATRA		1 (4.00%)
1 ABVD[1], BEACOPP[2], autologous transplantation (BEAM[3]) and DHAP[4]	1 (4.35%)	
R-CHOP[5], autologous transplantation (BEAM) and R-DHAP[6]	1 (4.35%)	
Cytarabine with anthracycline and autologous transplantation (busulfan, cyclophosphamide)	1 (4.35%)	
Multi-chemotherapy ALL protocol, with dasatinib and blinatumumab	1 (4.35%)	
R-CHOP, autologous transplantation (BEAM), ICE[7] and PI3K inhibitor	1 (4.35%)	
Chlorambucil and FCR[8]	1 (4.35%)	
CHOP and DHAP	1 (4.35%)	
FC[9], alemtuzumab and alvocidib	1 (4.35%)	
ABVD, DHAP, ICE, brentuximab		1 (4.00%)
VIP[10], DHAP, autologous transplantation (BEAM); brentuximab, rituximab		1 (4.00%)
Multichemotherapy ALL protocol		2 (8.00%)
Bortezomib, thalidomide, dexamethasone, autologous transplantation (melphalan), carfilzomib, lenalidomide		1 (4.00%)
DHAP, CHOP, SMILE, alemtuzumab, nelarabine		1 (4.00%)
**Immunosuppression**	**6 (26.10%)**	**1 (4.00%)**
Alemtuzumab	2 (8.70%)	
Cyclosporine	2 (8.70%)	
Anti-thymocyte globulin (ATG)	1 (4.35%)	
Cyclosporine and ATG	1 (4.35%)	
Not defined		1 (4.00%)
**Other**	**2 (8.70%)**	

Hydroxyurea	1 (4.35%)	
Hydroxyurea and Ruxolutinib	1 (4.35%)	
**No previous therapy**	**1 (4.35%)**	**4 (16.00%)**

ABDV[1]- doxorubicin hydrochloride (Adriamycin), bleomycin sulfate, vinblastine sulfate, and dacarbazine; BEACOOP[2]-bleomycin sulfate, etoposide phosphate, doxorubicin hydrochloride (Adriamycin), cyclophosphamide, vincristine sulfate (Oncovin), procarbazine hydrochloride, and prednisone; BEAM[3]- armustine, etoposide, cytarabine, and melphalan; (R-)DHAP[4,6]-(rituximab), dexamethasone, high-dose cytarabine, cisplatin; (R)-CHOP[5]-(rituximab), cyclophosphamide, doxorubicin hydrochloride, vincristine sulfate, and prednisone; ICE[7]-ifosfamide, carboplatin, and etoposide; FC(R)[8,9]-fludarabine, cyclophosphamide, (rituximab); VIP[10]-etoposide, ifosfamide, and cisplatin; SMILE[11]- dexamethasone, methotrexate, ifosfamide, L-asparaginase, and etoposide
