# Supplementary material for: Viral Infections in HSCT Recipients with Post-Transplant Lymphoproliferative Disorder: The Role of Torque Teno Virus as a Marker of Immune Functions
Source: Microorganisms. 2025 Feb 2;13(2):326. doi: 10.3390/microorganisms13020326 (PMC11857982; doi:10.3390/microorganisms13020326)
Supplement: Supplementary file 1 [file microorganisms-13-00326-s001.zip › Table S2.pdf]

Supplementary Table S2. Viral DNA found in plasma samples of PTLD-HSCT and Non-PTLD-HSCT group.

Patient	Timepoint	Viral DNA detected [in log10 copies/ml]				
		EBV	CMV	HHV-6	TTV	ADV
PTLD-HSCT 8	T0				4.35	
	T1	3.70			4.93	
	T2		2.70		2.71	
PTLD-HSCT 15	T0				3.80	
	T1				5.09	4.17
	T2				6.43	
PTLD-HSCT 17	T0				4.07	
	T1				4.63	
	T2			3.70	5.56	
PTLD-HSCT 18	T0			3.54	7.54	
	T1			2.50	8.36	
	T2				8.75	
PTLD-HSCT 19	T0				5.09	
	T1		3.60		6.72	
	T2	3.10			7.95	
PTLD-HSCT 23	T0				4.66	
	T1	2.18			8.36	
	T2	1.91			8.39	
Non-PTLD-HSCT 18	T0				3.07	
	T1				5.19	4.24
	T2				2.59	
Non-PTLD-HSCT 63	T0				3.36	
	T1		3.37		8.54	
	T2		2.68		8.36	
Non-PTLD-HSCT 65	T0		4.13		3.93	
	T1				5.92	
	T2				3.90	
Non-PTLD-HSCT 82	T0				4.09	
	T1			1.30	5.80	
	T2				5.74	
