# Supplementary material for: Viral Infections in HSCT Recipients with Post-Transplant Lymphoproliferative Disorder: The Role of Torque Teno Virus as a Marker of Immune Functions
Source: Microorganisms. 2025 Feb 2;13(2):326. doi: 10.3390/microorganisms13020326 (PMC11857982; doi:10.3390/microorganisms13020326)
Supplement: Supplementary file 1 [file microorganisms-13-00326-s001.zip › Table S3.pdf]

Supplementary Table S3. Multiple factors influencing TTV DNA load in PTLD patients after HSCT

**Patient**	**Timepoint after HSCT**					
	**T0**		**T1**		**T2**	
	**Mean [log_{10} copies/ml]**	**p-value**	**Mean [log_{10} copies/ml]**	**p-value**	**Mean [log_{10} copies/ml]**	**p-value**
**Age**						
<25 vs 26-55 years	3.99 vs 4.78	0.98	7.07 vs 6.41	0.99	6.61 vs 7.69	0.99
<25 vs >56 years	3.99 vs 4.38	>0.99	7.07 vs 5.95	0.92	6.61 vs 5.86	0.99
26-55 vs >56 years	4.78 vs 4.38	0.99	6.41 vs 5.95	0.99	7.69 vs 5.86	0.19
**Gender**						
Female vs Male	4.31 vs 4.87	0.65	6.01 vs 6.46	0.88	6.18 vs 7.17	0.53
**CMV status**						
CMV D-/R- vs CMV D+/R-	3.51 vs 5.05	0.94	4.28 vs 5.91	0.93	5.79 vs 6.49	>0.99
CMV D-/R- vs CMV D-/R+	3.51 vs 5.03	0.99	4.28 vs 6.30	0.99	5.79 vs 5.27	>0.99
CMV D-/R- vs CMV D+/R+	3.51 vs 4.36	NA	4.28 vs 7.43	NA	5.79 vs 8.04	NA
CMV D+/R- vs CMV D-/R+	5.05 vs 5.03	>0.99	5.91 vs 6.30	>0.99	6.49 vs 5.27	>0.99
CMV D+/R- vs CMV D+/R+	5.05 vs 4.36	NA	5.91 vs 7.43	NA	6.49 vs 8.04	NA
CMV D-/R+ vs CMV D+/R+	5.03 vs 4.36	NA	6.30 vs 7.43	NA	5.27 vs 8.04	NA
**CMV mismatch**						
Match vs mismatch	3.72 vs 5.04	0.17	5.07 vs 6.04	0.77	6.35 vs 6.08	0.99
**Co-infections**						
Presence vs Absence	7.54 vs 4.47	NA	6.69 vs 6.12	0.89	7.30 vs 6.11	0.89
**HPC source**						
HPC-A vs HPC-M	4.61 vs 4.58	>0.99	6.36 vs 5.82	0.87	7.09 vs 5.28	0.44
**HSCT subtype**						
MUD vs MRD	4.45 vs 4.39	>0.99	6.35 vs 6.25	>0.99	6.71 vs 5.59	>0.99
MUD vs Haplo-id	4.45 vs 5.23	0.99	6.35 vs 6.10	>0.99	6.71 vs 7.28	>0.99
MUD vs UCB	4.45 vs 3.79	NA	6.35 vs 5.09	NA	6.71 vs 6.43	NA
MRD vs Haplo-id	4.39 vs 5.23	>0.99	6.25 vs 6.10	>0.99	5.59 vs 7.28	>0.99
MRD vs UCB	4.39 vs 3.79	NA	6.25 vs 5.09	NA	5.59 vs 6.43	NA
Haplo-id vs UCB	5.23 vs 3.79	NA	6.11 vs 5.09	NA	7.28 vs 6.43	NA
**Underlying disorder type**						
Lymphoid vs myeloid malignancies	4.68 vs 4.86	0.99	5.70 vs 6.45	0.77	6.67 vs 6.61	0.99
**Ablative conditioning**						
No vs Yes	4.44 vs 5.00	0.79	6.33 vs 6.04	0.97	6.61 vs 6.90	0.98
**PTLD classification**						
Non Destructive vs Destructive	**3.54 vs 4.95**	**0.01**	**4.76 vs 6.43**	**0.03**	5.91 vs 6.79	0.84
**Outcome**						
Alive vs Deceased	**5.33 vs 3.94**	**0.03**	6.78 vs 5.75	0.32	**7.74 vs 5.74**	**0.02**

CMV status: D (Donor), R (recipient); HPC (Hematopoietic progenitor cells): HPC-A (Hematopoietic progenitor cells from apheresis), HPC-M (Hematopoietic progenitor cells from bone marrow); HSCT subtype: MUD (matched-unrelated-donor), MRD (matched-related-donor), haplo-id (Haploidentical hematopoietic stem cell transplantation), UCB (Umbilical cord blood); PTLD classification: ND (non-destructive), D (destructive); Survival: A (alive), D (Deceased).
