# Supplementary material for: Viral Infections in HSCT Recipients with Post-Transplant Lymphoproliferative Disorder: The Role of Torque Teno Virus as a Marker of Immune Functions
Source: Microorganisms. 2025 Feb 2;13(2):326. doi: 10.3390/microorganisms13020326 (PMC11857982; doi:10.3390/microorganisms13020326)
Supplement: Supplementary file 1 [file microorganisms-13-00326-s001.zip › Table S4.pdf]

Supplementary Table S4. Multiple factors influencing TTV DNA load in Non-PTLD patients after HSCT

Patient	Timepoint after HSCT					
	T0		T1		T2	
	Mean [log_{10} copies/ml]	p-value	Mean [log_{10} copies/ml]	p-value	Mean [log_{10} copies/ml]	p-value
**Age**						
<25 vs 26-55 years	3.46 vs 3.47	>0.99	7.03 vs 4.11	0.51	4.56 vs 3.51	0.94
<25 vs >56 years	3.46 vs 3.29	>0.99	7.03 vs 5.01	0.79	4.56 vs 3.76	0.99
26-55 vs >56 years	3.47 vs 3.29	>0.99	4.11 vs 5.01	0.71	3.51 vs 3.76	>0.99
**Gender**						
Female vs Male	3.33 vs 3.42	0.99	5.25 vs 4.63	0.73	3.98 vs 3.61	0.94
**CMV status**						
CMV D-/R- vs CMV D+/R-	3.68 vs 4.82	0.99	4.17 vs 5.10	0.97	3.50 vs 4.51	0.99
CMV D-/R- vs CMV D-/R+	3.68 vs 3.82	>0.99	4.17 vs 4.35	>0.99	3.50 vs 3.45	>0.99
CMV D-/R- vs CMV D+/R+	3.68 vs 2.56	0.72	4.17 vs 5.36	0.95	3.50 vs 3.75	>0.99
CMV D+/R- vs CMV D-/R+	4.82 vs 3.82	0.99	5.10 vs 4.35	0.99	4.51 vs 3.45	0.99
CMV D+/R- vs CMV D+/R+	4.82 vs 2.56	0.73	5.10 vs 5.36	>0.99	4.51 vs 3.75	>0.99
CMV D-/R+ vs CMV D+/R+	3.82 vs 2.56	0.96	4.35 vs 5.36	0.99	3.45 vs 3.75	>0.99
**CMV mismatch**						
Match vs mismatch	3.06 vs 3.90	0.49	4.80 vs 4.48	0.93	3.64 vs 3.45	0.97
**Co-infections**						
Presence vs Absence	3.93 vs 3.37	NA	6.51 vs 4.63	0.49	8.36 vs 3.55	NA
**HPC source**						
HPC-A vs HPC-M	3.38 vs 3.44	0.99	4.81 vs 5.21	0.99	3.69 vs 4.14	0.97
**HSCT subtype**						
MUD vs MRD	3.48 vs 3.20	>0.99	4.95 vs 4.68	>0.99	3.83 vs 3.65	>0.99
MUD vs Haplo-id	3.48 vs 3.74	0.99	4.95 vs 5.18	>0.99	3.83 vs 3.80	>0.99
MRD vs Haplo-id	3.20 vs 3.74	0.99	4.68 vs 5.18	0.99	3.65 vs 3.80	>0.99
**Underlying disorder type**						
Lymphoid vs myeloid malignancies	3.09 vs 3.36	0.98	5.89 vs 4.50	0.49	4.36 vs 3.52	0.42
**Ablative conditioning**						
No vs Yes	3.36 vs 3.42	0.99	4.34 vs 5.78	0.07	3.39 vs 4.38	0.39
**Outcome**						
Alive vs Deceased	3.29 vs 3.52	0.96	4.99 vs 4.68	0.94	3.90 vs 3.55	0.91

CMV status: D (Donor), R (recipient); HPC (Hematopoietic progenitor cells): HPC-A (Hematopoietic progenitor cells from apheresis), HPC-M (Hematopoietic progenitor cells from bone marrow); HSCT subtype: MUD (matched-unrelated-donor), MRD (matched-related-donor), haplo-id (Haploidentical hematopoietic stem cell transplantation); Survival: A (alive), D (Deceased).
