# Supplementary material for: Viral Infections in HSCT Recipients with Post-Transplant Lymphoproliferative Disorder: The Role of Torque Teno Virus as a Marker of Immune Functions
Source: Microorganisms. 2025 Feb 2;13(2):326. doi: 10.3390/microorganisms13020326 (PMC11857982; doi:10.3390/microorganisms13020326)
Supplement: Supplementary file 1 [file microorganisms-13-00326-s001.zip › Table S5.pdf]

Supplementary Table S5. Multiple factors influencing TTV DNA load in HSCT recipients

Patient	Timepoint after HSCT					
	T0		T1		T2	
	Mean [$\log_{10}$ copies/ml]	p-value	Mean [$\log_{10}$ copies/ml]	p-value	Mean [$\log_{10}$ copies/ml]	p-value
**Age**						
<25 vs 26-55 years	4.13 vs 4.09	>0.99	6.85 vs 5.02	0.33	5.38 vs 5.50	>0.99
<25 vs >56 years	4.13 vs 3.81	0.99	6.85 vs 5.46	0.48	5.38 vs 4.76	0.99
26-55 vs >56 years	4.09 vs 3.81	0.99	5.20 vs 5.46	0.99	5.50 vs 4.76	0.95
**Gender**						
Female vs Male	3.87 vs 4.04	0.95	5.67 vs 5.42	0.94	5.19 vs 5.14	0.99
**CMV status**						
CMV D-/R- vs CMV D+/R-	3.63 vs 4.91	0.64	4.20 vs 5.50	0.48	4.19 vs 5.50	0.93
CMV D-/R- vs CMV D-/R+	3.63 vs 4.12	>0.99	4.20 vs 4.83	0.99	4.19 vs 3.90	>0.99
CMV D-/R- vs CMV D+/R+	3.63 vs 2.76	0.86	4.20 vs 5.59	0.71	4.19 vs 4.22	>0.99
CMV D+/R- vs CMV D-/R+	4.94 vs 4.12	0.99	5.50 vs 4.83	0.99	5.50 vs 3.90	0.85
CMV D+/R- vs CMV D+/R+	4.94 vs 2.76	0.12	5.50 vs 5.59	>0.99	5.50 vs 4.23	0.99
CMV D-/R+ vs CMV D+/R+	4.12 vs 2.76	0.76	4.83 vs 5.59	0.99	3.90 vs 4.23	>0.99
**CMV mismatch**						
Match vs mismatch	3.22 vs 4.39	0.06	4.86 vs 5.15	0.93	4.21 vs 4.58	0.93
**Co-infections**						
Presence vs Absence	5.73 vs 3.89	0.86	6.62 vs 5.30	0.16	7.56 vs 4.94	0.059
**HPC source**						
HPC-A vs HPC-M	3.93 vs 4.16	0.95	5.51 vs 5.59	0.99	5.22 vs 4.86	0.96
**HSCT subtype**						
MUD vs MRD	4.06 vs 3.38	0.99	5.78 vs 4.92	0.93	5.54 vs 3.95	0.33
MUD vs Haplo-id	4.06 vs 4.76	0.99	5.78 vs 5.71	>0.99	5.54 vs 5.78	>0.99
MUD vs UCB	4.06 vs 3.79	NA	5.78 vs 5.09	NA	5.54 vs 6.43	NA
MRD vs Haplo-id	3.38 vs 4.76	0.89	4.92 vs 5.71	0.99	3.95 vs 5.78	0.87
MRD vs UCB	3.38 vs 3.79	NA	4.92 vs 5.09	NA	3.95 vs 6.43	NA
Haplo-id vs UCB	4.76 vs 3.79	NA	5.71 vs 5.09	NA	5.78 vs 6.43	NA
**Underlying disorder type**						
Lymphoid vs myeloid malignancies	3.89 vs 3.98	0.99	5.80 vs 5.31	0.83	5.51 vs 4.80	0.67
**Ablative conditioning**						
No vs Yes	3.90 vs 4.12	0.95	5.34 vs 5.89	0.61	5.00 vs 5.48	0.86
**Outcome**						
Alive vs Deceased	4.19 vs 3.74	0.61	5.78 vs 5.24	0.62	5.59 vs 4.69	0.42

CMV status: D (Donor), R (recipient); HPC (Hematopoietic progenitor cells): HPC-A (Hematopoietic progenitor cells from apheresis), HPC-M (Hematopoietic progenitor cells from bone marrow); HSCT subtype: MUD (matched-unrelated-donor), MRD (matched-related-donor), haplo-id (Haploidentical hematopoietic stem cell transplantation), UCB (Umbilical cord blood); Survival: A (alive), D (Deceased).
